# Innervation of the developing kidney *in vivo* and *in vitro*

**DOI:** 10.1242/bio.060001

**Published:** 2023-07-31

**Authors:** Julia Tarnick, Mona Elhendawi, Ian Holland, Ziyuan Chang, Jamie A. Davies

**Affiliations:** Deanery of Biomedical Science, University of Edinburgh, Edinburgh EH8 9XD, UK

**Keywords:** Kidney, Development, Innervation, Neurons

## Abstract

Within the adult kidney, renal neurites can be observed alongside the arteries where they play a role in regulating blood flow. However, their role and localization during development has so far not been described in detail. In other tissues, such as the skin of developing limb buds, neurons play an important role during arterial differentiation. Here, we aim to investigate whether renal nerves could potentially carry out a similar role during arterial development in the mouse kidney. In order to do so, we used whole-mount immunofluorescence staining to identify whether the timing of neuronal innervation correlates with the recruitment of arterial smooth muscle cells. Our results show that neurites innervate the kidney between day 13.5 and 14.5 of development, arriving after the recruitment of smooth muscle actin-positive cells to the renal arteries.

## Teaser

**Summary:** In the developing mouse kidney, neurons first invade the kidney between day 13 and 14 of embryonic development and follow the course of the maturing arteries.

## INTRODUCTION

Renal innervation is involved in the regulation of systemic blood pressure. Over-activity of sympathetic renal neurons can lead to hypertension (Osborn and Foss, 2017). Consequently, the removal of the nerves that lie along the renal artery can be used to treat hypertension (De Jager et al., 2016). There is currently no evidence of the negative effect of the denervation of the renal artery on renal function (Sanders et al., 2017), so one might conclude that extrinsic innervation is not essential for the arterial function of the adult kidney. However, renal neurons may play an important role during development.

Within the skin of the developing limb, sensory nerves control the differentiation of blood vessels into arteries (Li et al., 2013). In other tissues, such as the spinal cord, neurons have been shown to control the positioning of blood vessels (Himmels et al., 2017). It is therefore reasonable to ask whether they do so in the developing kidney. Logically, if neurites are required for positioning and/or differentiation of arteries, they must be present at the sites of future arteries before this differentiation has taken place. A first step toward testing the hypothesis that neurons control renal arterial development is therefore to study the relative locations and times of the appearance of neurites and developing arteries.

In mice, the first blood vessels invade the kidney around embryonic day (E) 11.5 of development and grow in close proximity to the collecting duct network (Munro, 2017). Around E13.5 the vessels start to establish a hierarchical network, the hierarchy visible as changes in diameter of what will be larger vessels, as well as the upregulation of the arterial marker Connexin 40 in the interlobular arteries (Daniel, 2018). During the following 24 h of development, smooth muscle cells differentiate from FoxD1-expressing stromal progenitors in the periphery and migrate towards the blood vessels to form the arterial smooth muscle cell lining (Hurtado, 2015). Whether neurons are present in the kidney at this stage has, to the best of our knowledge, not been investigated.

The innervation of adult kidneys has been studied extensively. Nerve fibres originating primarily from the aorticorenal suprarenal ganglia (Torres et al., 2021) lie mainly along the blood vessels, where they control contraction and dilation of the vessel (Bradford, 1889; Harman and Davies, 1948). The afferent fibres extend into the glomerulus (Harman and Davies, 1948; Wu et al., 2021). The majority of nerve endings are found in the adventitia of vessels and epithelia of the renal pelvis (Harman and Davies, 1948).

Early studies of human fetal kidneys found neurons mainly in proximity with blood vessels, but also around the medial and juxtaglomerular region of the nephron (Zimmermann, 1972). A recent study by Mompeó et al. (2019) describes the early innervation of the human kidney in detail. Consistent with previous studies (Mitchell, 1953; Tiniakos et al., 2004), the authors noticed the presence of neurons alongside the renal arteries. However, according to the authors, the innervation of the kidney coincides with the formation of the renal veins but occurs 2 weeks after differentiation of the renal arteries (Mompeo et al., 2019). Similar to those observed in mice (Torres et al., 2021), the nerves in human kidneys express tyrosine hydroxylase (TH) (Tiniakos et al., 2004).

In rats, nerves positive for calcitonin gene-related peptide can be detected from embryonic day E16.5 (Liu and Barajas, 1993). The afferent rat neurites were observed along the blood vessels that enter the kidney as well as along the ureter and within the mesenchyme (Liu and Barajas, 1993). Efferent neuropeptide Y-positive nerves were predominantly found along the renal arteries and, to a lesser extent, along the renal pelvis and ureter (Liu and Barajas, 1993).

In the kidneys of E11 mouse embryos, a micro-ganglion has been observed at the connection between the ureter and Wolffian duct (Sariola et al., 1988). To the best of our knowledge, the innervation of the murine kidney during later stages of murine embryonic development has not yet been described. In this study, by staining mouse embryonic kidneys between E14.5 and E17.5 of development, we examined the relative times and positions of neuronal and arterial development. We found that arteries appear prior to innervation, the neurites extending later along the paths of existing arteries. We also found that both arteries and neurites degenerate when kidney rudiments are removed from their embryos and placed in culture.

## RESULTS

### Neurites invade the kidney between E13.5 and E14.5 following the course of arterial smooth muscle cells

**Fig. 1.**
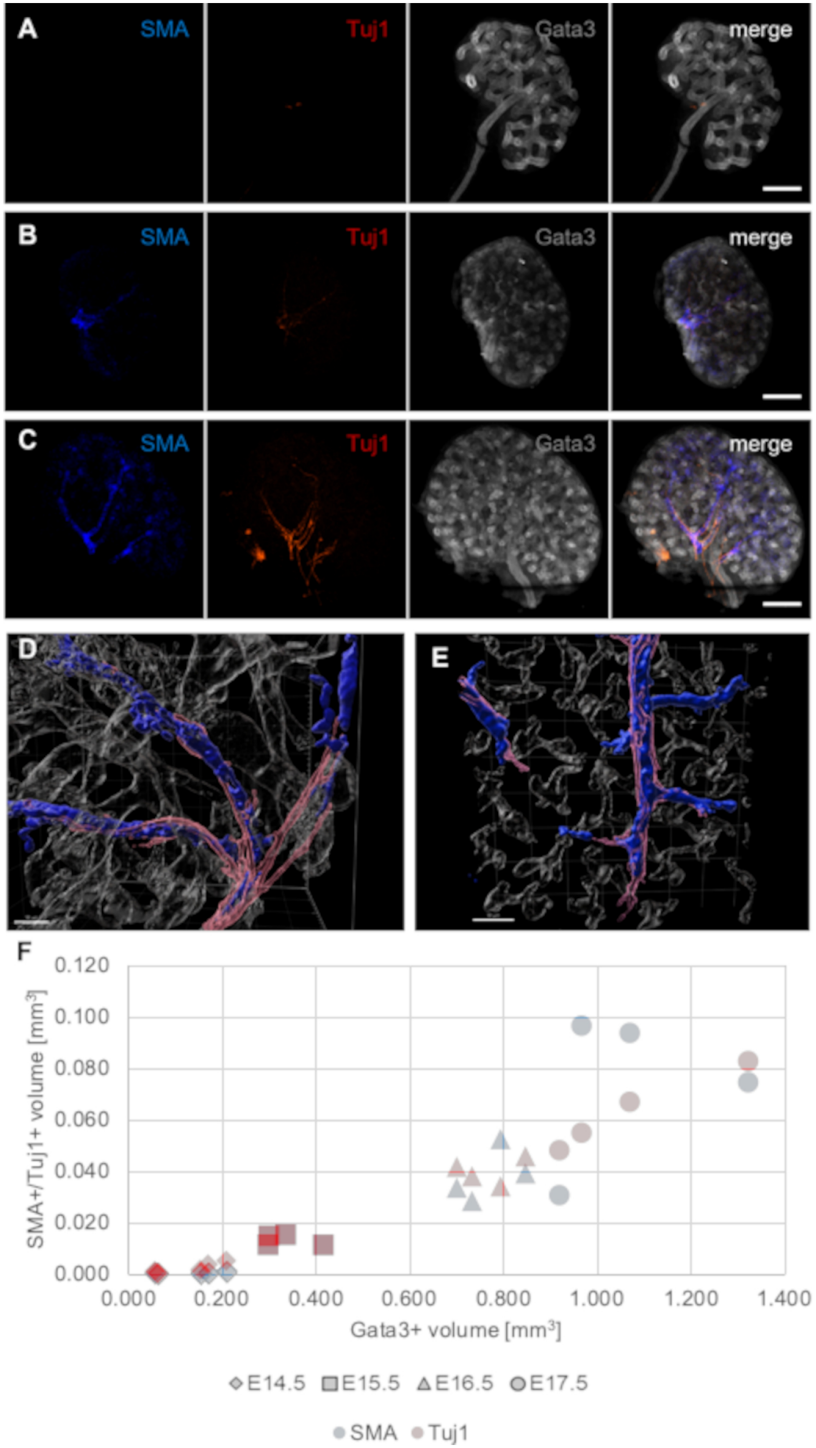
**Neurons invade the kidneys between E13.5 and E14.5 following the path of arterial smooth muscle cells**. (A) Neurites were absent in isolated E13.5 kidneys. (B,C) E14.5 kidneys showed variable degrees of smooth muscle cell coverage and innervation, and the amount of neurites closely correlated with the degree of vascular smooth muscle layer formation. Images in A-C are maximum projections of stacks representing approximately half the depth of each kidney. (D,E) 3D renderings of E14.5 (D) and E17.5 (E) kidneys from *z*-stacks 155 µm thick and with a step size of 5 µm reveal that neurites are found in close proximity and, to a large degree, in direct contact with SMA-positive arterial smooth muscle cells. (F) Quantification of the volume of SMA-positive (blue) and Tuj1-positive (red) structures relative to Gata3 in E14.5 to E17.5 kidney explants. The volumes of SMA and Tuj1 follow a similar trend throughout development. Scale bars: 200 µm (A-C); 50 µm (D,E).

Within the adult kidney, neurites align along arteries where they regulate blood flow (Bradford, 1889; Harman and Davies, 1948). To obtain evidence on whether neurites might also be involved in the maturation of arteries in the kidney, we isolated developing mouse kidneys (the structure of which is summarized in Fig. 5) at a range of ages between E13.5 and E17.5 and stained them for the early neuronal marker Tuj1 to show the location of developing neurites. We also stained them for markers of smooth muscles, which are a feature of arterial development: these markers were smooth muscle actin (SMA) (Saga et al., 1999) and calponin 1 (Calp1 or Cnn1) (di Gioia et al., 2000). In addition, we stained for markers of the general anatomy of the organ such as Gata3 (Labastie et al., 1995) (Fig. 1; Fig. S1) and collagen IV (Kuroda et al., 1998) (Fig. S2).

Isolated E13.5 kidneys displayed variable degrees of maturity with respect to smooth muscles, with smooth muscle cells already present in some, but by no means all, explants. Fig. 1A, not showing smooth muscle cells, is typical; Fig. S2 shows an example where some muscle cells were present. We did not observe any neurons within E13.5 kidneys (Fig. 1A, representing one of four kidneys examined; Fig. S2), but two out of 11 explants contained neurons outside the kidney itself, along the ureter. The presence of SMA-positive cells in some of the explants (Fig. S2) indicates that the recruitment of smooth muscle cells to developing arteries was initiated prior of arrival of Tuj1-positive renal neurons (Fig. 1A; Fig. S1). In E14.5 kidneys, vascular smooth muscle cells were consistently present, but the extent to which the smooth muscle cell lining was formed varied between samples (Fig. 1B,C, representing five kidneys examined). During later stages of embryonic kidney development (Figs S1 and S2, four kidneys examined at E16.5 and four at E17.5), neurites were found to align along all major SMA-positive arteries. To identify how closely the neurites aligned with smooth muscle cells, we performed three-dimensional (3D) renderings of E14.5 and E17.5 kidneys (Fig. 1D,E). The rendering showed that neurites were very close to and probably in direct contact with the smooth muscle cells. No neurons were observed alongside SMA-negative blood vessels. Occasionally, neurites appeared to extend slightly further along the blood vessel than the expression of the smooth muscle cell marker Calp1, which is expressed slightly later within renal arterial smooth muscle cells than SMA (Fig. S3). The close correlation between vascular smooth muscle cell recruitment and innervation was further highlighted by a similar growth pattern when plotted against the volume of Gata3-positive structures (Fig. 1F).

### Neurites were not found in close proximity to developing nephrons but seen in contact with a small subset of Bowman’s capsules in E17.5 kidneys

**Fig. 2.**
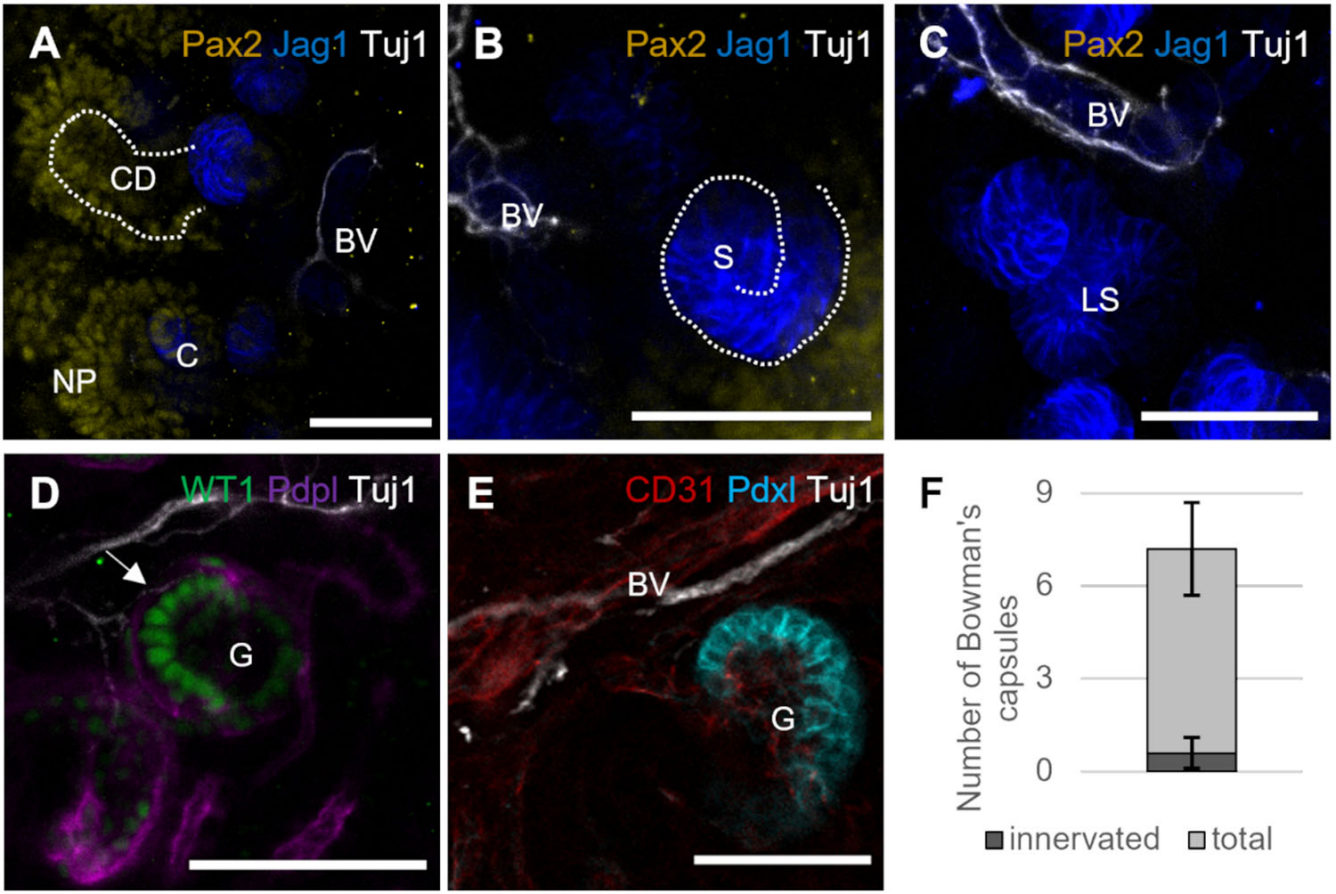
**Neurites were not associated with developing nephrons but were found in direct contact with a small subset of Bowman's capsules of E17.5 kidneys.** (A) Staining of the nephrogenic zone for Pax2, Tuj1 and Jagged1 (Jag1) shows neurites in contact with nearby blood vessels (BV, identified by morphology and weak Jag1 expression) but absent from the regions occupied by nephron progenitor cells (NP) and collecting duct tips (CD). We did not observe any contact with comma-shaped (C) early nephrons. (B) Neurites continued to be found alongside blood vessels (BV), but not found in association with S-shaped nephrons (S). (C) A sample image of a late S-stage (LS) nephron. No contact with neurites was observed for this stage of nephron development. (D) Single-plane image of a Bowman’s capsule stained for WT1 and podoplanin (Pdpl) shows a Tuj1-positive neurite forming contact with the podocyte layer around the glomerulus (G; arrow). A-C show *z*-stacks of three to five sections; D is a single optical section. (E) Representative single-plane image of the glomerulus (G) stained for CD31, surrounded by podcalyxin (Pdxl)-positive podocytes. In this sample, neurites were detected alongside nearby blood vessels (BV) but not found to be in contact with the glomerular capillaries. (F) Quantification of the number of Bowman's capsules found in direct contact with neurites after manual count of five *z*-stacks taken from three biological replicates. Error bars show s.d. The Bowman's capsules were analysed frame-by-frame throughout their entire thickness to ensure that no connections were missed. Bowman's capsules that were not fully captured by the *z*-stack were excluded from analysis. All scale bars: 50 µm.

Our results suggest that neurites remain in close contact with the developing arteries. However, to rule out any possible association with developing nephrons, we imaged the nephrogenic zone of three E17.5 kidneys at higher magnification (Fig. 2).

When we co-stained kidney rudiments with the neurite marker Tuj1, Pax2 (a transcription factor expressed at high levels in nephron progenitor cells, in nephrons and to a lower extent in the collecting ducts) and Jagged1 (or Jag1, a marker for the medial region of developing nephrons), we found no contact between neurites and nephron progenitor cells (Fig. 2A, representing five kidneys examined) or developing nephrons (Fig. 2A-C).

To identify whether neurites would contact the Bowman's capsule, we co-stained E17.5 kidneys with WT1 and podoplanin (Pdpn) (Fig. 2D, representing five kidneys examined), as well as with the endothelial marker CD31 (or Pecam1) and the podocyte marker podocalyxin (Podxl) (Fig. 2E, representing three kidneys). Most Bowman's capsules, of 36 examined, showed no contact with neurites. We manually counted the Bowman's capsules within *z*-stacks, each containing five to ten Bowman's capsules, taken from three different kidneys and assessed. An average of 7% of Bowman's capsules were found to be in contact with neurites (Fig. 2D,F).

### Renal neurons express the dopaminergic marker TH

Previous studies (Torres et al., 2021; Wu et al., 2021) have shown that the renal neurons of adult mice express the dopaminergic marker TH. To identify at what stage renal neurons gain their dopaminergic character, we stained E14.5 and E16.5 kidneys for TH and Tuj1.

**Fig. 3.**
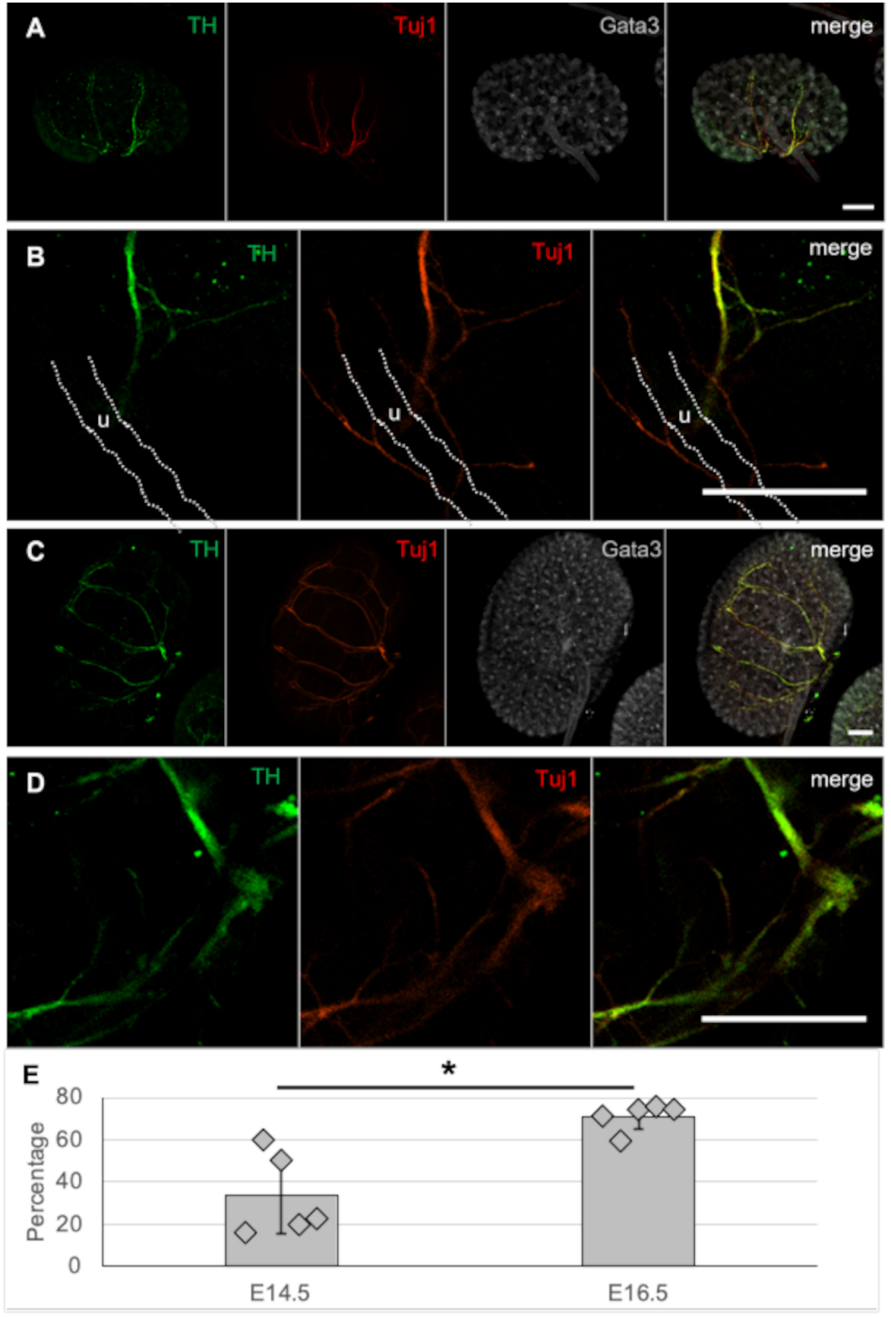
**Developing renal neurons express TH.** (A) E14.5 mouse kidneys stained for the early-stage pan-neuronal marker Tuj1 and dopaminergic linage marker TH reveal co-expression within most perivascular neurons, whereas peri-ureter neurons stained negative for TH. (B) Higher-magnification image of A showing the TH-negative neurites surrounding the ureter (u). (C) In E16.5 kidneys, all Tuj1-positive neurons visible in this image co-expressed TH, which is also visible at higher magnification (D). Images are maximum-projections of *z*-stacks covering approximately half the depth of each kidney: Scale bars: 200 µm. (E) Quantification of Tuj1-positive structures that co-express TH showed a significant increase from 34% at E14.5 to 71% at E16.5. *P<0.004, according to two-tailed unpaired Student's *t*-test. Error bars show s.d.

At E14.5, most neurons along the renal artery expressed TH, whereas neurons along the ureter were negative for TH (Fig. 3A,B, representing five kidneys examined). In E16.5 kidneys, most Tuj1-positive neurons also expressed TH (Fig. 3C,D, representing four kidneys examined). To further characterize the degree of co-expression at E14.5 and E16.5, we quantified the percentage of Tuj1-positive structures that also express TH using the ImageJ plugin JaCoP (https://imagej.net/plugins/jacop). Whereas at E14.5, 34% of Tuj1-positive structures expressed TH, the degree of increased co-expression was statistically significant (*P*<0.01) to 71% at E16.5 (Fig. 3E, representing five E14.5 and five E16.5 kidneys). The measured degree of co-localization was slightly lower than what we had expected by visual inspection of the stained explants, which suggested a close to 100% overlap: this difference is probably caused by slight differences in the subcellular localization of TH and Tuj1.

Published studies in adult kidneys (Torres et al., 2021) have revealed that a subset of renal neurons is negative for TH but can be detected by injection of the retrograde viral tracer, pseudorabies virus (PRV)-152. Tuj1 is commonly regarded as an early-stage pan-neuronal marker (Memberg and Hall, 1995), suggesting that the TH-negative neurons that were detected by Torres et al. (2021) using viral tracing might develop or invade the kidney at a later stage, perhaps during postnatal development.

### Cultured E11.5 kidneys contain neurons, some of which express TH

Kidneys grown in organ culture provide a good opportunity to study and manipulate renal development *in vitro* without the need for invasive animal experiments. Therefore, we aimed to characterize how neurons develop in renal explant cultures.

**Fig. 4.**
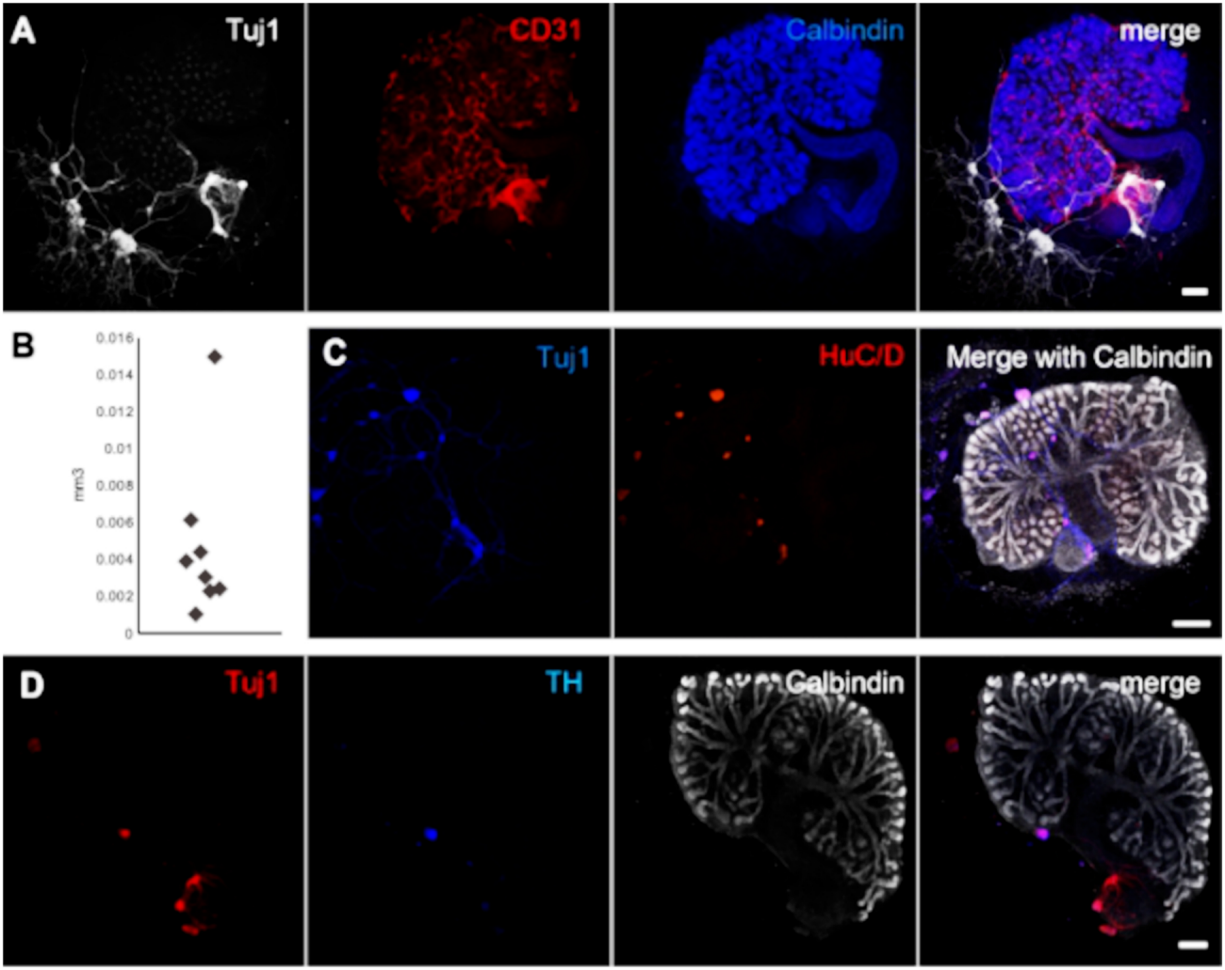
**Neurons develop in E11.5 kidney explants**. (A) 7-day-cultured E11.5 kidneys contain variable Tuj1-positive neurons. The neurites were not found to be preferentially associated with the blood vessels of the explants. (B) Quantification of total volume of Tuj1-positive structures in mm^3^ per explant. (C) Staining of cultured kidney explants for the neuronal body marker HuC/D. (D) The explants contained dense aggregates of Tuj1-positive neurons that expressed TH, suggesting differentiation of renal neurons *in vitro*. Images are maximum-projection *z*-stacks representing the entire depth of the kidney. Scale bars: 200 µm (A,D); 300 µm (C).

Consistent with previous studies (Sariola et al., 1988), we found neurites to be present in kidney rudiments removed from their embryos at E11.5 and cultured for 7 days (Fig. 4A). The extent of neural colonization varied greatly between explants (Fig. 4B, representing eight samples). The neurons were mostly found in the peripheral areas of the renal explants, with few axon-like processes growing into the kidneys. Furthermore, the explants showed dense aggregates of Tuj1-positive structures that expressed the neuron body marker HuC/D (Desmet et al., 2014) (Fig. 4C). The ureters were closely surrounded by neurons (Fig. 4).

To identify whether neurons that had already invaded the kidneys could maintain their natural localization, we isolated kidneys at E14.5 and cultured them for up to 24 h, fixing sets of explants after 8, 16 and 24 h (Fig. S4). Cultured E14.5 explants progressively lost the expression of SMA in perivascular smooth muscle cells, whereas the cells surrounding the ureter maintained SMA expression. We observed many neurons displaying a fragmented morphology, suggesting that they were dying (Fig. S2). Although the majority of neurons displayed the fragmented morphology, we also observed intact neurons at all culture time points analysed.

To verify whether renal neurons could differentiate in explant cultures, we stained 7-day-cultured kidneys for TH. Most Tuj1-positive structures were negative for TH. However, we did observe some dense spherical aggregates that stained positive for TH, suggesting that neuronal differentiation is possible, but limited in renal explant cultures (Fig. 4D).

During natural development, TH expression is present in axons at E14.5 and onward (Fig. 3). However, in E14.5 kidneys that had been cultured for 8 to 32 h, TH was detectable in round aggregates but not in elongated axon-shaped structures (Fig. S3), suggesting that differentiated neurons may lose their identity in culture.

## DISCUSSION

Whole-mount immunofluorescence staining revealed that, within developing mouse kidneys, innervation occurs after the recruitment of arterial smooth muscle cells and that, during late embryonic development (E17.5), most neurons within the kidney express the dopaminergic lineage marker TH. The paucity of TH-negative neurons in E17.5 kidneys was surprising as in adult kidneys, many TH-negative neurons can be detected through the injection of a retrograde viral tracer (Torres et al., 2021). Tuj1 is considered an early-stage, pan-neuronal marker, which suggests that either TH-negative neurons invade the kidney at a later stage or some of the neurons that initially express TH lose their expression during later development. The staining of postnatal kidneys could clarify whether the expression of TH is lost in a subset of neurons at a later stage or whether a second wave of innervation takes place during postnatal development.

**Fig. 5.**
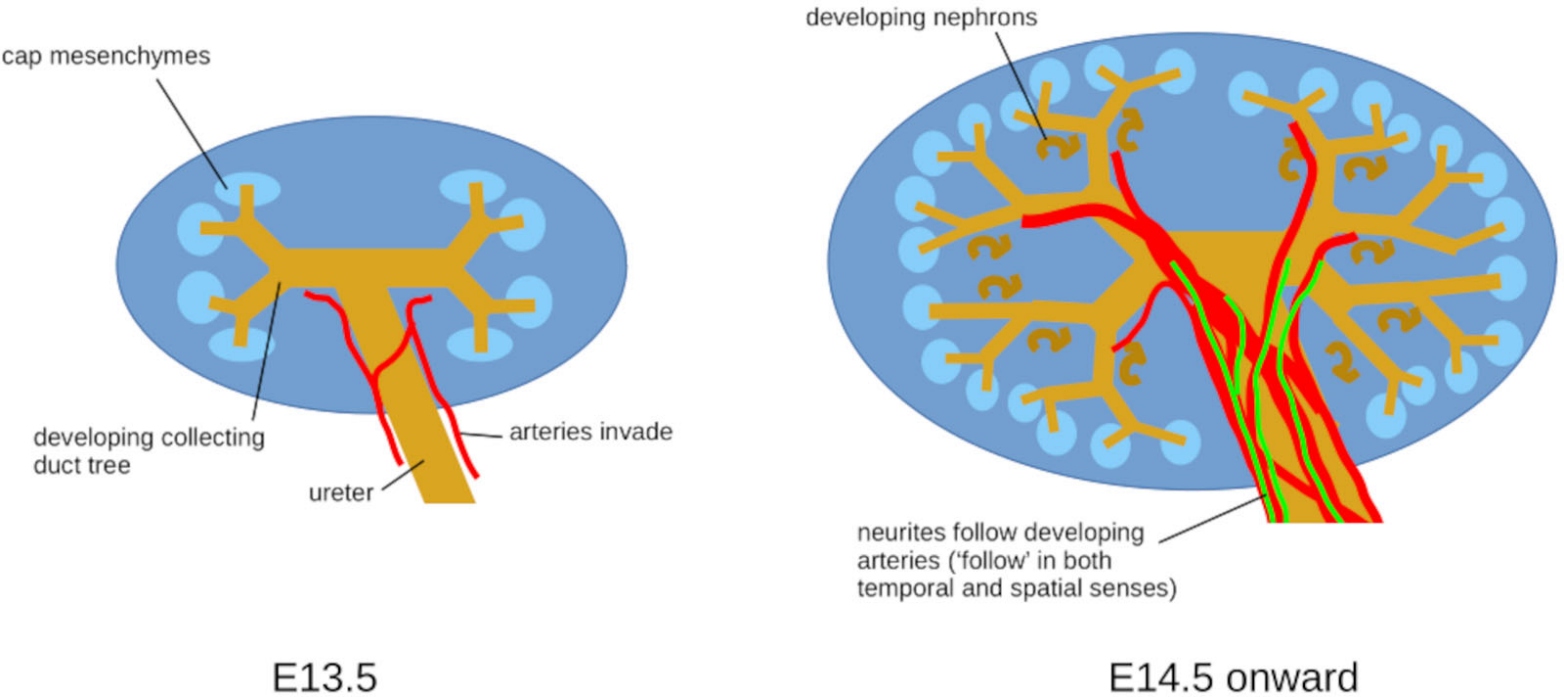
**Summary diagram of the findings in this paper.** Arteries arrive in the kidney first and neurites extend later, mainly along arteries. This relative timing indicates that neurites cannot be necessary for artery formation (although it does not rule out more subtle influences on later artery development/stability).

The timing of renal innervation suggests that, within the kidney, in contrast to the skin of the developing limb bud, neurons are not required to initiate arterial development (Fig. 5). However, renal neurons could play a role during the maturation and maintenance of arterial smooth muscle cells.

The innervation of the renal arteries appeared to correlate closely with the expression of the smooth muscle cell marker calponin 1, which, in the vascular smooth muscle cells, was upregulated later than smooth muscle actin. Calponin 1 is assumed to play a role in regulating smooth muscle cell contractility and maturation, although it is not essential for either process (Liu and Jin, 2016). Its expression, and that of other smooth muscle cell markers, can be induced by serum release factor (SRF) and TGFβ (Choo et al., 2022; Werth et al., 2010). SRF and TGFβ can in turn be activated in response to norepinephrine, a product of dopaminergic neurons (Fisher and Absher, 1995; Hennenberg et al., 2012; Paradis et al., 1996), supporting the hypothesis that neurons may play a role in smooth muscle cell maturation in the renal arteries. Further evidence for a role of neurons in supporting smooth muscle cell growth is that cultured E14.5 kidneys display a progressive loss of smooth muscle cells, subsequent to the downregulation of TH and fragmentation of renal neurons. This is, however, a correlation and not an indication of cause; further experiments are needed to identify whether neurons play a role during arterial smooth muscle cell maturation. Kidney explant cultures may be a useful tool for this as they resemble the organ morphology relatively closely and could be treated with compounds such as norepinephrine to identify whether this could upregulate or maintain the expression of smooth muscle cell markers such as calponin 1 *in vitro*.

Our results give an overview over the time course of the innervation of the embryonic kidney. Although we can refute the hypothesis that neurons are required to initiate smooth muscle cell recruitment, the co-occurrence of neurons with late smooth muscle cell markers raises questions about their potential role in promoting smooth muscle cell maturation.

## MATERIALS AND METHODS

### Kidney culture

Embryonic kidneys were obtained from timed-mated CD1 mice, the morning of the discovery of the vaginal plug being considered E0.5 of development. The pregnant mothers were culled by a licenced member of the Biomedical Veterinary Service using methods approved by Schedule 1 of the UK Animals (Scientific Procedures) Act 1986.

Kidney rudiments obtained by microdissection of embryos were either fixed directly in 4% paraformaldehyde (PFA) in phosphate-buffered saline (PBS) or, for explant experiments, cultured at the air-liquid interface by placing them on a polyester transwell insert (six-well, 0.4 µm pore size, Scientific Laboratory Supplies, 3450). The bottom well was filled with Minimum Essential Eagle Medium (Merck, 5650) supplemented with 10% fetal bovine serum (Gibco) and 1% penicillin/streptomycin (Gibco).

### Immunofluorescence staining, clearing and quantification

Freshly isolated kidneys were fixed in 4% PFA for 1 h at room temperature. Subsequently, the kidneys were washed three times with PBS and dehydrated in graded alcohols (20%, 40%, 60%, 80% and 100% methanol). Cultured kidneys were fixed directly in 100% methanol because PFA fixation frequently led to detachment of the kidney from the transwell membrane and thereby to loss of samples.

After dehydration, the samples were treated with 5% hydrogen peroxide in methanol for 4 h to bleach autofluorescent compounds. Following bleaching, the samples were rehydrated and washed three times with PBS. Washed samples were permeabilized for 4 h in PBS containing 0.2% Triton X-100, 20% dimethyl sulfoxide (DMSO) and 300 mM glycine. Subsequently, the samples were blocked overnight in PBS containing 3% donkey serum, 10% DMSO and 0.2% Triton X-100. After blocking, the primary antibodies (Table S1) were diluted in PBS containing 5% DMSO and 3% donkey serum and incubated with the samples for 24 to 72 h at room temperature. Unbound primary antibodies were removed by four washes with PBS prior to overnight incubation with the secondary antibodies (Table S1), which were diluted in PBS containing 3% donkey serum. Then, the samples were washed four times with PBS and dehydrated stepwise in methanol. The dehydrated kidneys were incubated overnight in 100% ethyl cinnamate and then transferred into an 18-well µ-slide (ibidi) for imaging.

Imaging was performed using a Nikon A1 confocal microscope. *Ex vivo* kidneys were generally presented as a maximum-projection of a *z*-stack capturing approximately half of the depth of the kidney, and cultured kidneys (which are much thinner) as a maximum-projection of a *z*-stack representing the entire thickness. Some images (e.g. Fig. 2D,E) are single optical sections, and this has been indicated in the figure legend. Imaging settings were optimized for each antibody individually but were then held constant for all images of that antibody in each set of experiments. Brightness and contrast of final images were adjusted slightly using ImageJ to improve visibility in print, and these changes were carried out for the entire image (experiments, controls, etc.). No individual structures have been altered within any image. Tuj1-positive structures were quantified as described previously (https://visikol.com/blog/2018/11/29/blog-post-loading-and-measurement-of-volumes-in-3d-confocal-image-stacks-with-imagej/). 3D renderings were reconstructed using Imaris software (Bitplane).

## Supplementary Material

10.1242/biolopen.060001_sup1 Supplementary information
