# Supplementary material for: Innervation of the developing kidney in vivo and in vitro
Source: Biol Open. 2023 Jul 31;12(8):bio060001. doi: 10.1242/bio.060001 (PMC10411870; doi:10.1242/bio.060001)
Supplement: Supplementary information [file biolopen-12-060001-s1.pdf]

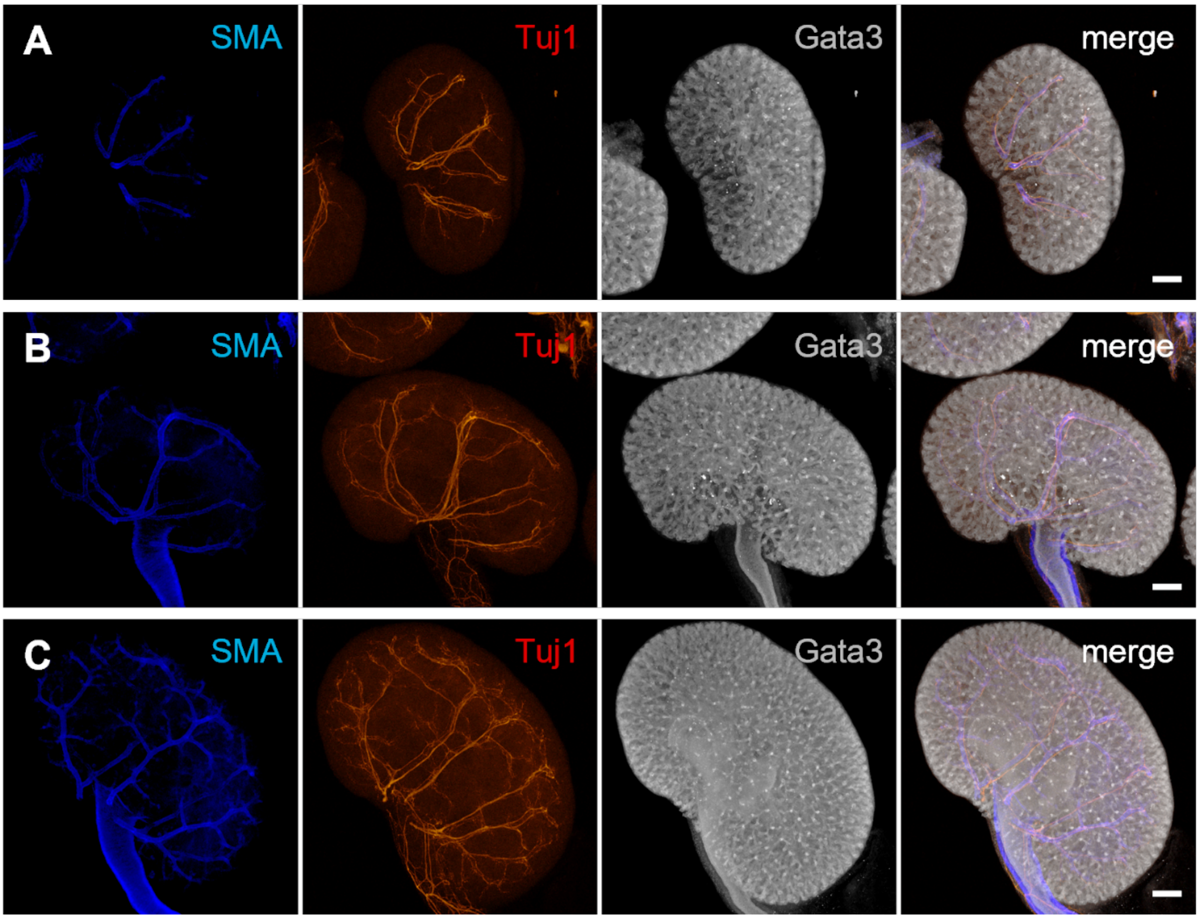

Fig. S1. Staining of E15.5 (A), E16.5 (B) and E17.5 (C) kidneys shows that renal neurites continue to grow along the SMA-positive blood vessels. Images are maximum projections of z-stacks covering about half of the depth of the kidney. Scale bars: 200 μm.

Biology Open • Supplementary information

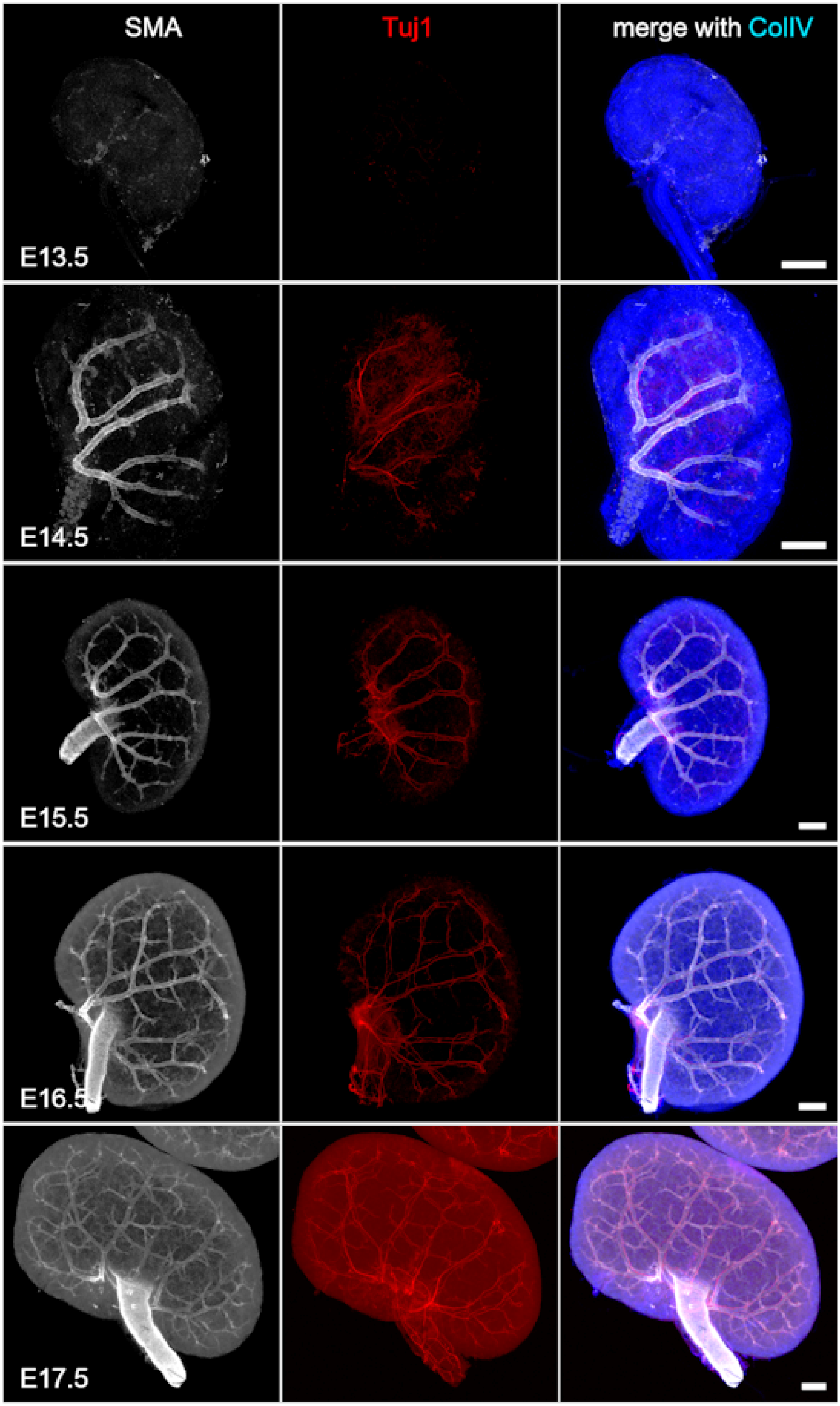

Fig. S2. Staining of isolated E13.5 to E17.5 kidneys for smooth muscle cell marker smooth muscle actin (SMA) early neurite marker Tuj1 and collagen IV (collIV) shows that neurites are absent in E13.5 kidneys while SMA is weakly expressed. During later stages neurites are seen in close proximity to SMA-positive vascular smooth muscle cells. Images are maximum projections of z-stacks covering about half of the depth of the kidney. Scale bars: 200 μm.

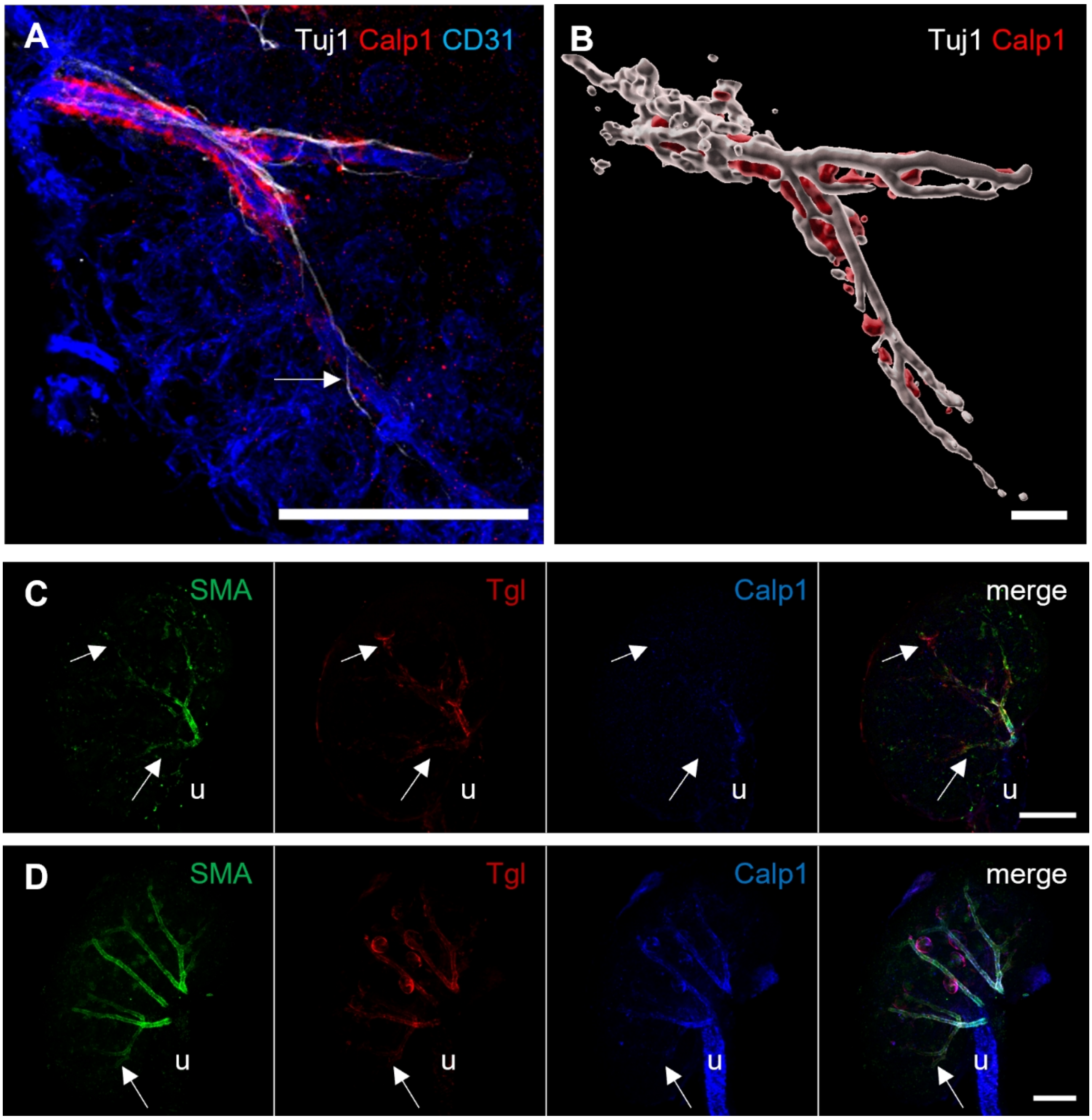

Fig. S3. Neurites extend further along blood vessels than the expression of late smooth muscle cell marker Calponin 1A: Staining for Tuj1 and Calponin 1 showed that the neurite tips extended further along the vessels than the expression of Calp1 was observed. Scale bar: 200 µm. B: 3D rendering of Calp1 and Tuj1 stained kidney enables better visualization of Calp1 positive section of the blood vessel in position to the neurite tips. To verify whether Calp1 is expressed later during vascular smooth muscle cell expression. E13.5 (C) and E14.5 (D) kidneys were stained for smooth muscle actin (SMA, green), Transgelin (TGL, red) and Calponin 1 (Calp1, blue) SMA was found to be most widely expressed along the renal arteries, followed by Transgelin. In contrast, the expression of Calponin 1 was initially limited to the section of the arteries near the ureter (u) and absent from the distal ends (arrows). Images are maximum projections of z-stacks covering about half of the depth of the kidney. Scale bar: 200 µm.

Biology Open • Supplementary information

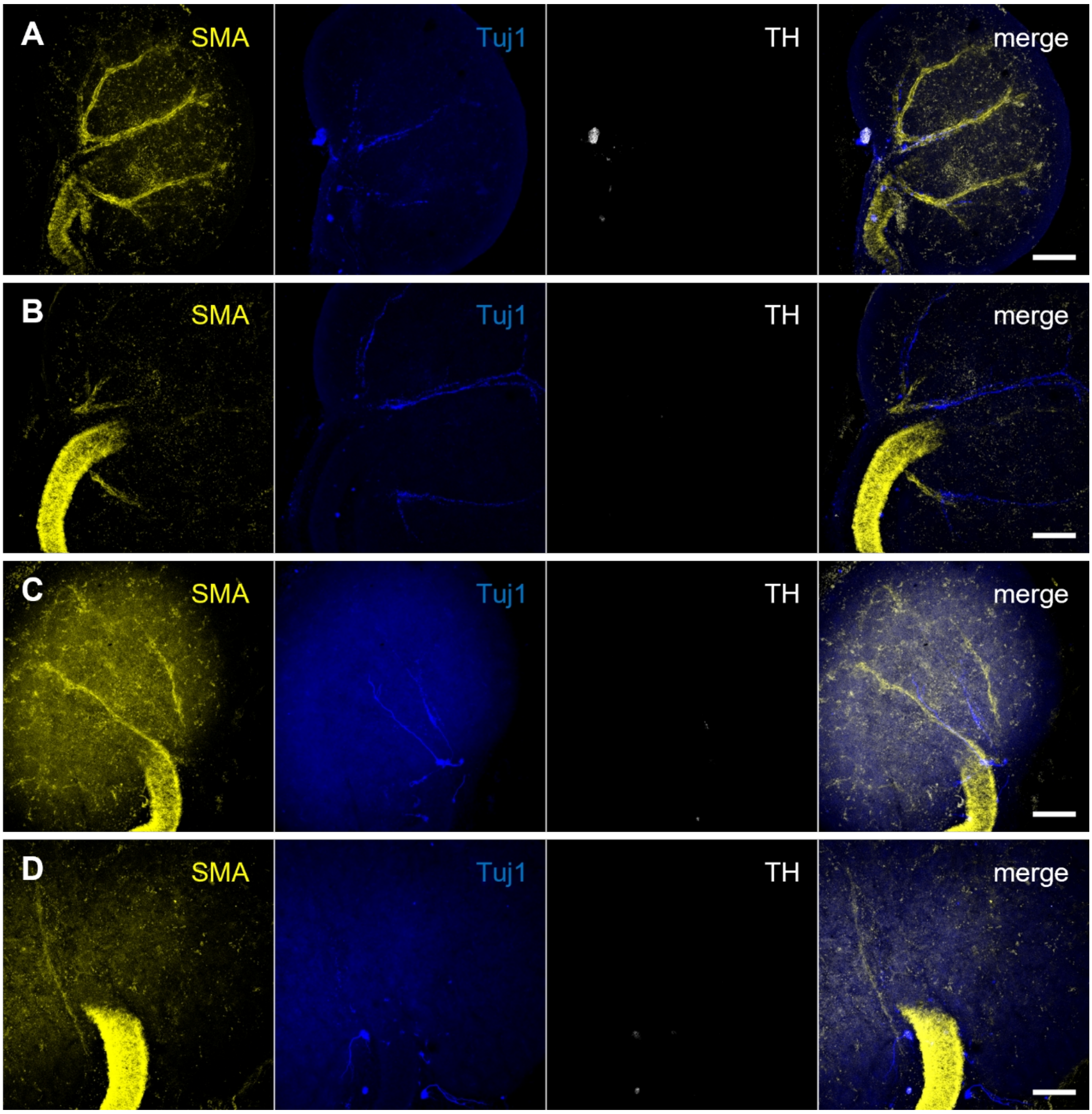

Fig. S4. Neurites in cultured E14.5 kidneys are negative for tyrosine hydroxylase (TH, grey) and often display a fragmented morphology. E14.5 kidneys were isolated and cultured for 8h (A), 16h (B), 24h (C) and 32h (D) followed by staining for smooth muscle actin (SMA, yellow), Tuj1 (blue) and TH (grey). During the culture a progressive loss of arterial smooth muscle cells is visible, while the expression of SMA remains high in the ureter. TH was not detectable in elongated Tuj1 positive neurites, but occasionally found in small spherical aggregates. The majority of Tuj1 positive neurites displayed a fragmented morphology, however a subset of continuous neurites was at all observed culture time points. Images are maximum projections of z-stacks covering the depth of the kidney. Scale bars: 200 µm.

Table S1. List of antibodies. All Antibodies were used in a 1:200 dilution

Primary antibody	Supplier	Catalogue number
**Calbindin**	Novus	NBP2-50028
**Calponin 1**	Abcam	ab46794
**CD31**	R&D	AF3628
**Collagen IV**	Merck	AB769
**Cytokeratin 8**	DHSB	TROMA-I
**Jagged 1**	R&D	AF599
**Podocalyxin**	R&D	MAB1556
**Smooth muscle actin-FITC**	Merck	3777
**Transgelin**	R&D	AF7886
**Tuj1**	Biolegend	801201
**Tuj1-AlexaFluor594**	Biolegend	801207
**Tyrosine Hydroxylase**	Merck	AB152
**Secondary Antibody**	**Supplier**	**Catalogue Number**
**Chicken-anti-rat AlexaFluor594**	Invitrogen	A21471
**Donkey-anti-chicken-FITC**	Abcam	ab63507
**Donkey-anti-goat AlexaFluor647**	Invitrogen	A21447
**Donkey-anti-mouse AlexaFluor555**	Invitrogen	A31570
**Donkey-anti-rabbit AlexaFluor488**	Invitrogen	A21206
**Donkey-anti-rabbit AlexaFluor594**	Invitrogen	A21207
**Donkey-anti-rabbit AlexaFluor647**	Invitrogen	A31573
**Donkey-anti-sheep AlexaFluor594**	Invitrogen	A11016

Biology Open • Supplementary information
